# Multi-site Vibration Signal Analysis for Estimating Post-Swallow Residue: A Pilot Study

**DOI:** 10.1007/s00455-026-10958-z

**Published:** 2026-05-16

**Authors:** Jee Hyun Suh, Jaewon Lee, Hyun Jin Kim, Jung‑Min Kim, Jinju Lee, Ju Seok Ryu

**Affiliations:** Department of Rehabilitation Medicine, Seoul National University Bundang Hospital, Seoul National University College of Medicine, 82 Gumi-ro 173 beon-gil, Bundang-gu, Seongnam-si, 13620 Gyeonggi-do South Korea

**Keywords:** Dysphagia, Pharyngeal residue, Acoustic signal analysis, Vallecular sinus, Harmonic-to-noise ratio

## Abstract

This pilot study investigated whether multi-site acoustic signal analysis could detect post-swallow residue and aimed to identify the most effective sensor location and parameters for detection. Three contact microphone-based acoustic sensors and one air-conduction-based conventional audio recorder were positioned at four anatomical sites: the jugular notch, near the pyriform sinus, near the vallecular sinus, and over the right deltoid muscle. Patients with dysphagia were recorded during pre-swallow, thin-fluid swallowing, and yogurt (YP) swallowing conditions. Acoustic parameters, including harmonic-to-noise ratio (HNR), noise-to-harmonic ratio, jitter, shimmer, and formant frequencies, were extracted from each recording. The videofluoroscopic swallowing study findings served as the reference standard for detecting pharyngeal residue. Pre-swallow and post-YP swallowing conditions yielded the most reliable acoustic indicators of post-swallow residue. Among the sensor locations, the right-lateral vallecular sinus sensor demonstrated the highest accuracy in reflecting pharyngeal retention. HNR emerged as the most robust single parameter, with high values paradoxically associated with the presence of residue. Multisite acoustic analysis, particularly HNR measurement from a vallecular sinus-placed sensor in the pre-swallow or post-YP swallowing state, may serve as a noninvasive and accessible method for detecting post-swallow residue in patients with dysphagia. Notably, the vallecular sensor demonstrated the highest diagnostic performance, suggesting that a simplified single-sensor approach may be feasible, although this requires validation in larger-scale studies. Largescale studies are needed to validate these findings and assess the feasibility of real-time dysphagia screening.

## Introduction

Dysphagia is a common symptom, and its incidence increases with age. The prevalence of dysphagia is around 40% among patients older than 60 years [1]. A study found that 63% of older adults who reported no subjective swallowing difficulties exhibited abnormalities in videofluoroscopic swallowing study (VFSS) [2]. VFSS is widely regarded as the gold standard for assessing dysphagia. During the procedure, patients swallow contrast media, enabling real-time visualization of the swallowing mechanism [3]. In addition to evaluating swallowing mechanics, VFSS enables the detection of penetration and aspiration events. Importantly, excessive pharyngeal residue in the valleculae beyond a certain threshold is associated with an increased risk of aspiration during subsequent swallowing [4]. However, VFSS can only be performed in facilities equipped with fluoroscopic imaging systems and the necessary technical infrastructure. In addition to the inherent risk of radiation exposure, the examination is not feasible for patients with unstable medical conditions or those unable to maintain the seated posture required for imaging. Furthermore, owing to time limitations, VFSS does not allow continuous or long-term monitoring of swallowing function.

To overcome these limitations, various noninvasive approaches have been explored. Voice analysis can be used to screen individuals at high risk of aspiration [5]. According to a systematic review, Doppler ultrasound demonstrated excellent diagnostic accuracy in distinguishing swallowing sounds, microphones showed good accuracy in detecting swallowing sounds in patients with dysphagia, and stethoscopes were found to be the most effective for screening purposes [6]. However, these studies have primarily focused on the detection of penetration and aspiration, with little attention given to the evaluation of post-swallow residue [7, 8]. Moreover, previous acoustic studies have predominantly relied on air-conduction microphones placed near the mouth, which are susceptible to environmental noise and may not adequately reflect subtle biomechanical changes within the pharyngeal structures. In addition, some studies have reported that voice changes are not consistently associated with post-swallow residue, highlighting the limitations of conventional voice-based approaches for residue detection [9].

To address these limitations, this study introduces a novel approach using contact microphone–based vibration sensors that directly capture tissue-conducted signals from anatomically relevant sites. Furthermore, this study systematically compares multiple anatomical sensor locations, including the vallecular and pyriform sinus regions, to identify the most effective site and acoustic parameters for detecting post-swallow residue.

The aim of this study was to investigate whether an air-conduction–based standard digital voice recorder and acoustic sensors based on contact microphones placed at the vallecula, pyriform sinus, and jugular notch can detect post-swallow residue and to identify which sensor parameters are most strongly correlated with residue.

## Methods

### Participants

This prospective study was conducted at the Department of Rehabilitation Medicine of a tertiary university hospital between April and November, 2023. The inclusion criteria were as follows: (i) patients 19 years or older who had undergone VFSS, (ii) those who exhibited symptoms of dysphagia, (iii) patients able to sustain phonation of “ah” for 5 s, and (iv) patients able to tolerate vibration sensors attached to the vallecula, pyriform sinus, and jugular notch. The exclusion criteria were as follows: (i) patients unable to follow one-step commands, (ii) those unable to phonate, (iii) those in whom sensor attachment was not feasible owing to skin hypersensitivity or related conditions, and (iv) those who did not undergo VFSS, voice recording, or vibration sensor assessments.

Medical information on sex, age, and underlying diseases was also collected. Written informed consent was obtained from all patients, and the study protocol was approved by the Institutional Review Board (B-2206-761-302).

### VFSS

All participants were evaluated using initial VFSS in a standardized manner [10, 11]. During VFSS, 3 mL of small-volume fluid (SF, International Dysphagia Diet Standardization Initiative [IDDSI] Level 0), 5 mL of large-volume fluid (LF, IDDSI Level 0), and 3 mL of yogurt (YP, IDDSI Level 2) were administered. The procedures were digitally recorded as video files for subsequent analysis. One physiatrist experienced in VFSS interpretation and dysphagia management evaluated the recordings. Post-swallow residue in the valleculae and pyriform sinuses was graded on a four-point scale: grade 0, no residue; grade 1, residue occupying less than 10% of the width of the respective sinus; grade 2, residue occupying 10% to less than 50% of the width; and grade 3, residue occupying more than 50% of the width, as visualized on the fluoroscopic images.

Pharyngeal residue was evaluated using the Normalized Residue Ratio Scale (NRRS) [12, 13] and calculated at the valleculae (NRRS_v) and pyriform sinuses (NRRS_p). The NRRS score was calculated as the ratio of the residue volume to the pharyngeal cavity, adjusted for individual cervical spine size [12]. The specific video frame used for the analysis corresponded to the moment when the hyoid bone descended to its lowest position after swallowing. A single physiatrist with over a decade of experience in interpreting VFSS performed all NRRS measurements.

### Acoustic Signal Analysis

**Fig. 1:**
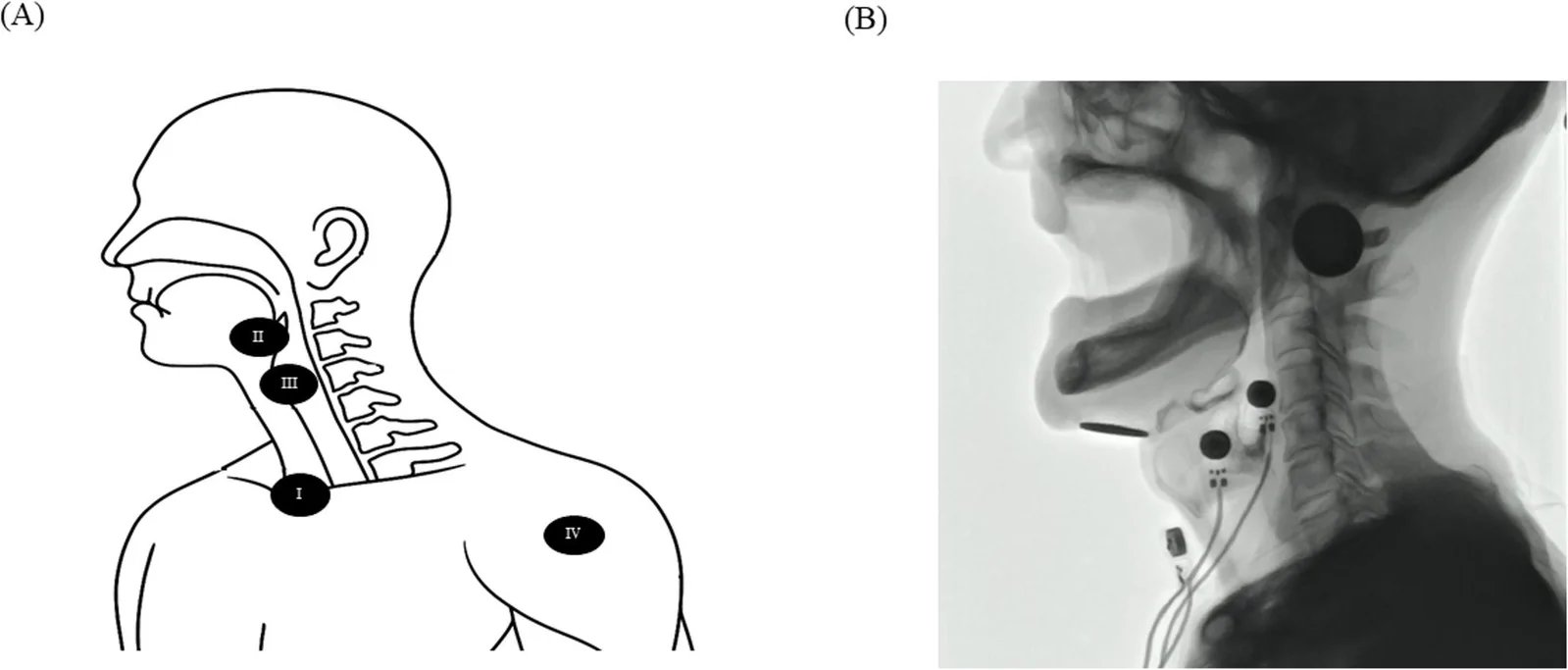
Locations of the acoustic sensor and the air-conduction-based standard digital voice recorder. (**a**) Schematic illustration of placement. (**b**) Actual placement confirmed during videofluoroscopic swallowing study (VFSS)

In this study, three vibration sensors based on contact microphones (C411L, AKG Acoustics, Austria) were used to detect body-surface vibrations, along with an air-conduction–based standard digital voice recorder (ICD-TX660, Sony Corporation, Japan) for conventional audio recording. The C411L (AKG Acoustics, Austria) is a vibration pickup contact condenser microphone designed to detect mechanical vibrations directly from the surface to which it is attached. Unlike traditional air-conduction microphones, this sensor captures sound through contact, making it particularly suitable for applications where airborne noise must be minimized. The C411L was used as a vibration sensor based on a contact microphone. The acoustic sensors were attached to the jugular notch area (Sensor I) and right lateral side of the valleculae (Sensor II) and pyriform sinuses (Sensor III), with placement guided by real-time fluoroscopic imaging during VFSS (Fig. 1). Additionally, an air-conduction–based standard digital voice recorder was positioned over the right deltoid area (Sensor IV) (Fig. 1).

Voice and vibration measurements were performed prior to VFSS and before ingestion of any food or liquid. Patients were instructed to sustain the phonation of “ah” for 5 s, which was used as the baseline value. Subsequently, after the ingestion of 3 mL of SF, 5 mL of LF, and 3 mL of YP, patients were asked to repeat the “ah” phonation for 5 s after each swallow.

### Data Preprocessing and Voice Variable Extraction

Voice data were standardized using a multistep preprocessing workflow. First, a Python-based spectral gating algorithm, which is a form of noise gate, was applied for noise reduction. Based on the statistical and sensitivity analyses, the threshold was set at 0.8. A mask was generated by comparing the signal spectrogram with the threshold. This mask was smoothed in both the frequency and time domains, applied to the spectrogram, and inverse-transformed to reconstruct the noise-reduced signal. Second, the Density-Based Spatial Clustering of Applications with Noise algorithm was used to detect spikes and outliers in the audio signal [14]. This algorithm identifies high-density point clusters and assigns points with markedly different characteristics to noise clusters [15]. Parameters were optimized through preliminary testing. For each audio clip, the epsilon was set between 0.3 and 0.5, and the minimum sample size was set between three and 15 [16]. Detected segments were verified by auditory review and removed when confirmed as artifacts. Finally, a silence-detection algorithm from the Python PyDub library was applied to remove nonspeech intervals [17].

Acoustic features were extracted from the preprocessed clips using the Python Parselmouth and SciPy libraries [18]. Various acoustic parameters, including maximal and average intensity, fundamental frequency (f0), standard deviation of f0, harmonic-to-noise ratio (HNR), noise-to-harmonic ratio (NHR), jitter, shimmer, and formants, were measured.

### Statistical Analysis

All statistical analyses were performed using SPSS software (version 22.0; SPSS Inc., Chicago, IL, USA). Statistical significance was set at *p* < 0.05. In this study, the data could not be assumed to follow a normal distribution owing to the small sample size. The Mann–Whitney U test was conducted to compare the acoustic parameters between the non-residue and residue groups. Kendall’s tau correlation analysis with bootstrapping was performed to examine the relation between post-swallow residue and acoustic voice parameters. Multivariate linear regression was used to identify the associations between the acoustic parameters and NRRS_v and NRRS_p.

## Results

### Clinical Characteristics of the Participants

Nineteen patients were included in this study. The mean age was 73.11 ± 13.87 years, and 13 were men. The most common underlying conditions were presbyphagia (36.8%) and cerebrovascular disease (31.6%). Residue was observed in 42.1% of patients, whereas 57.9% showed no residue. Aspiration was present in 47.4% of patients (Table 1).

**Table 1:** Demographic data

Total (*N* = 19)		Total	Residue Group	Non-residue Group
Age (years)		73.11 ± 13.87	75.00 ± 16.27	71.73 ± 12.48
Sex	Male	13	8	5
	Female	6	0	6
Underlying disease	Cerebrovascular diseases	6	3	3
	Parkinson disease	2	1	1
	Dementia	1	1	0
	Motor neuron disease	1	0	1
	Guillain–Barré syndrome	1	0	1
	Myopathy	1	0	1
	Presbyphagia	7	3	4
VFSS result	Normal	7	0	7
	Aspiration	4	0	4
	Aspiration with residue	5	5	0
	Residue	3	3	0
PAS	1	7	1	6
	2	0	0	0
	3	2	1	1
	4	1	1	0
	5	4	2	2
	6	1	1	0
	7	1	0	1
	8	3	2	1
Residue	Yes	8	8	0
	No	11	0	11
Vallecular residue	0	11	0	11
	1	7	7	0
	2	1	1	0
	3	0	0	0
Pyriformis residue	0	12	1	11
	1	6	6	0
	2	0	0	0
	3	1	1	0
NRRS_v		0.04 ± 0.07	0.04 ± 0.07	0
NRRS_p		0.08 ± 0.19	0.08 ± 0.19	0

### Comparison of Acoustic Voice Parameters between Residue and Non-Residue Groups

The results of the comparison between the residue and non-residue groups are presented in Table 2. Only acoustic parameters with statistically significant differences (*p* < 0.05) are reported.

**Table 2:** Comparison of acoustic voice parameters between residue and non-residue groups

	Residue Group	Non-residue Group	*p*-value
Pre(I) max dB	-13.37 ± 3.43	-16.38 ± 2.35	0.02*
Pre(II) average dB	-16.00 ± 1.86	-14.54 ± 2.39	0.04*
Pre(II) HNR	22.21 ± 4.49	12.91 ± 5.30	0.00*
Pre(II) NHR	0.01 ± 0.01	0.10 ± 0.13	0.01*
Pre(II) rap jitter	0.39 ± 0.25	0.84 ± 0.56	0.03*
Pre(II) ddp jitter	1.19 ± 0.76	2.53 ± 1.69	0.03*
Pre(II) local shimmer	6.25 ± 2.63	12.13 ± 5.19	0.01*
Pre(II) local dB shimmer	0.59 ± 0.23	1.09 ± 0.42	0.01*
Pre(II) apq5 shimmer	4.08 ± 1.75	8.27 ± 3.88	0.01*
Pre(II) apq11 shimmer	5.49 ± 2.49	11.12 ± 5.85	0.02*
Pre(II) dda shimmer	8.07 ± 2.76	17.03 ± 8.15	0.01*
SF(I) f0Hz	223.61 ± 35.88	185.97 ± 38.94	0.04*
SF(III) average dB	-26.42 ± 0.64	-27.00 ± 0.38	0.04*
LF(II) apq11 shimmer	6.09 ± 3.17	12.67 ± 5.68	0.03*
YP(I) st dev Hz	8.95 ± 4.75	22.41 ± 19.18	0.04*
YP(II) HNR	19.96 ± 6.78	12.69 ± 5.90	0.04*
YP(II) NHR	0.03 ± 0.04	0.11 ± 0.16	0.04*
YP(II) local jitter	0.83 ± 0.76	1.44 ± 1.01	0.04*
YP(II) rap jitter	0.42 ± 0.46	0.75 ± 0.61	0.03*
YP(II) ppq5 jitter	0.40 ± 0.36	0.79 ± 0.67	0.04*
YP(II) ddp jitter	1.27 ± 1.37	2.27 ± 1.85	0.03*
YP(II) local shimmer	7.42 ± 3.99	13.69 ± 5.64	0.03*
YP(II) local dB shimmer	0.70 ± 0.36	1.19 ± 0.49	0.04*
YP(II) apq3 shimmer	3.39 ± 1.82	5.46 ± 2.04	0.04*
YP(II) apq11 shimmer	6.57 ± 3.53	12.35 ± 5.33	0.01*
YP(II) dda shimmmer	10.16 ± 5.45	16.39 ± 6.13	0.04*

Before swallowing, significant group differences were observed in the amplitude detected by Sensor I and in the amplitude, HNR, NHR, jitter, and shimmer measured by Sensor II. After the SF trial, significant differences were observed in the f0 measured by Sensor I and the amplitude measured by Sensor III.

Following the LF trial, the apq11 shimmer measured by Sensor II showed a significant difference.

After the YP trial, the standard deviation of f0 measured by Sensor I and the HNR, NHR, jitter, and shimmer measured by Sensor II were significantly different between the groups.

In summary, the pre-swallow condition and YP trial yielded the most significant group differences, and the region around the vallecular sinus (Sensor II) appeared to be the most sensitive location for detecting residue-related acoustic changes. Among the acoustic parameters, HNR, NHR, jitter, and shimmer emerged as meaningful indicators associated with post-swallow residue.

### Kendall’s Tau Correlation Analysis with Bootstrapping

Given the nonparametric nature of the data, Kendall’s tau correlation analysis with bootstrapping was performed to evaluate the associations between acoustic parameters and total residue, NRRS_v, and NRRS_p. Significant correlations were observed between acoustic parameters, including dB level, HNR, NHR, jitter, and shimmer, measured in the vallecular sinus area before dietary intake, showing moderate associations with Kendall’s tau coefficients ranging from 0.42 to 0.56. Following SF intake, the f0 measured near the jugular notch and dB level measured near the pyriform sinus demonstrated significant associations. After ingestion of LF, shimmer measured in the vallecular sinus area showed a significant correlation. Similarly, after YP intake, HNR, NHR, jitter, and shimmer measured in the vallecular sinus exhibited moderate associations, with Kendall’s tau coefficients ranging from 0.43 to 0.45 (Table 3).

**Table 3:** Correlation between post-swallow residue and acoustic voice parameters

	Residual_sum		NRRS_v		NRRS_p	
	r	p	r	p	r	p
Pre(I) max dB	0.49	0.01*	0.45	0.01*	0.31	0.09
Pre(II) average dB	0.44	0.03*	0.39	0.04*	0.41	0.03*
Pre(II) HNR	0.56	0.01*	0.52	0.01*	0.46	0.02*
Pre(II) NHR	-0.52	0.01*	-0.54	0.00*	-0.41	0.03*
Pre(II) rap jitter	-0.42	0.04*	-0.36	0.06	-0.25	0.19
Pre(II) ddp jitter	-0.42	0.04*	-0.36	0.06	-0.25	0.19
Pre(II) local shimmer	-0.45	0.02*	-0.59	0.00*	-0.32	0.09
Pre(II) local dB shimmer	-0.49	0.01*	-0.60	0.00*	-0.34	0.08
Pre(II) apq5 shimmer	-0.43	0.03*	-0.57	0.00*	-0.30	0.11
Pre(II) apq11 shimmer	-0.42	0.04*	-0.54	0.00*	-0.27	0.16
Pre(II) dda shimmer	-0.47	0.02*	-0.55	0.00*	-0.32	0.09
SF(I) f0Hz	0.44	0.03*	0.45	0.02*	0.31	0.12
SF(III) average dB	0.49	0.02*	0.41	0.04*	0.51	0.01
LF(II) apq11 shimmer	-0.40	0.06	-0.47	0.02*	-0.28	0.17
YP(I) st dev Hz	-0.43	0.04*	-0.37	0.06	-0.22	0.26
YP(II) HNR	0.43	0.04*	0.42	0.03*	0.28	0.16
YP(II) NHR	-0.43	0.04*	-0.42	0.03*	-0.28	0.16
YP(II) local jitter	-0.43	0.04*	-0.35	0.07	-0.24	0.23
YP(II) rap jitter	-0.45	0.03*	-0.39	0.05	-0.28	0.16
YP(II) ppq5 jitter	-0.43	0.04*	-0.37	0.06	-0.26	0.19
YP(II) ddp jitter	-0.45	0.03*	-0.39	0.04*	-0.28	0.16
YP(II) local shimmer	-0.45	0.03*	-0.46	0.04*	-0.28	0.16
YP(II) local dB shimmer	-0.43	0.04*	-0.44	0.02*	-0.26	0.19
YP(II) apq3 shimmer	-0.43	0.04*	-0.39	0.04*	-0.24	0.23
YP(II) apq11 shimmer	-0.55	0.01*	-0.53	0.01*	-0.35	0.07
YP(II) dda shimmmer	-0.43	0.04*	-0.39	0.04*	-0.24	0.23

### Multivariate Linear Regression Analysis

Multivariate linear regression analysis was conducted to identify the acoustic parameters associated with NRRS_v and NRRS_p observed in VFSS. The resulting regression model was as follows:

  

$$\rm NRRS_v = -0.12 + 0.009(Pre(II)HNR)$$

Multivariate linear regression revealed no significant predictors of NRRS_p among the acoustic parameters. These findings suggest that specific acoustic parameters derived from voice analysis may have predictive value in estimating vallecular residue after swallowing.

## Discussion

In this pilot study, we investigated whether multi-site acoustic signal analysis could reflect post-swallow residue and aimed to identify the sensor location that best captured this information. The results demonstrated that the pre-swallow condition and YP trial provided the most reliable acoustic indicators of post-swallow residue. The acoustic sensors attached to the vallecular sinus region most accurately reflected the residue. Additionally, among the various signal features analyzed, HNR was the most reliable indicator. Unlike previous studies that primarily relied on air-conduction microphones to detect aspiration-related voice changes, this study applied contact microphone–based vibration sensors that directly capture tissue-conducted signals, thereby reducing susceptibility to environmental noise and enabling detection of subtle biomechanical changes associated with residue. Furthermore, this study is the first to systematically compare multiple anatomically distinct sensor locations for residue detection and to identify HNR measured at the vallecular region as a potential key acoustic biomarker. Collectively, these findings suggest that analyzing HNR measured by an acoustic sensor attached to the vallecular area, either in the pre-swallow state or after YP swallowing, may be the most appropriate approach for detecting post-swallow residue. To the best of our knowledge, this is the first study to analyze post-swallow residue using acoustic sensors placed at multiple anatomical locations and across various dietary consistencies.

The results of this study showed that the pre-swallow condition and YP trial provided the most reliable acoustic indicators of post-swallow residue. In the pre-swallow condition, patients with dysphagia may have pooled secretions, such as sputum, in the pharyngeal space [19]. These secretions correspond to IDDSI levels 1–2, and their presence may have contributed to pharyngeal retention, thereby influencing the acoustic measures [11]. In the case of YP, classified as IDDSI level 2, the likelihood of retention was higher than that of thin fluids (IDDSI level 0) [20]. This may explain why the pre- and post-YP swallowing conditions more accurately reflected the degree of residue in this study.

In this study, three vibration sensors based on contact microphones and an air-conduction-based conventional audio recorder were used. The sensors were positioned at the jugular notch, near the pyriform and vallecular sinuses, and over the right deltoid. Among these, the sensor placed on the right lateral side of the vallecular sinus (Sensor II) most accurately reflected the presence of pharyngeal retention. This finding can be explained as follows: anatomically, the vallecular sinus is located superior to the epiglottis, whereas the pyriform sinus is located inferior [21]. In this study, participants were instructed to produce the vowel /a/ after swallowing. According to previous studies, during the articulation of the vowel /a/, the opening between the pharynx and epiglottis maintains a general shape similar to that observed in the production of pharyngeal consonants but with a larger aperture [22]. However, when residue is present in the vallecular sinus, the weight of the retained material may exert slight pressure on the epiglottis, leading to passive displacement. This subtle movement of the epiglottis can be detected using a sensor placed near the vallecular sinus. Furthermore, the damping effect caused by vallecular residue may transmit minute vibrations to the surrounding tissues, which are captured more sensitively by the vallecular sensor than by the other sensors [23]. These combined factors may explain why the sensor positioned at the vallecular region (Sensor II) most accurately reflected the degree of pharyngeal retention among the four sensors used.

Although a multi-sensor setup was employed in this pilot study for exploratory purposes, our results consistently demonstrated that the sensor placed at the vallecular region showed the highest diagnostic performance. This finding suggests that a simplified single-sensor configuration targeting the vallecular area may be sufficient for detecting post-swallow residue, thereby enhancing clinical feasibility, although this should be validated in larger-scale studies. Future studies should focus on further validating this single-sensor approach and exploring its applicability in practical bedside or wearable settings.

In this study, among the various signal features analyzed, HNR was the most reliable indicator for detecting post-swallow residue. In addition, NHR, jitter, and shimmer were associated with post-swallow residue. Typically, a higher HNR is associated with clear and stable voice quality, whereas increased jitter indicates irregular vocal-fold vibration, often perceived as a shaky or unstable voice. Similarly, high shimmer reflects amplitude instability, commonly resulting in a harsh, hoarse, or breathy voice with perceptible variation in loudness. However, in this study, the acoustic parameters recorded from the vallecular sinus area paradoxically showed high HNR and low NHR, jitter, and shimmer values in the presence of pharyngeal residue. This finding is contrary to conventional expectations and suggests that when the sensor is attached near the vallecular area, the pooled residue may act as a biological damper, leading to a damping effect on local sound vibrations. This damping may suppress the transmission of high-frequency noise components and irregularities in the acoustic signal, thereby resulting in reduced NHR, jitter, and shimmer values despite the presence of pharyngeal residue.

From a clinical perspective, these findings highlight the potential of vallecular sinus-based acoustic monitoring as a noninvasive and radiation-free method for detecting pharyngeal residue. Such an approach could be implemented as an adjunctive screening tool in settings where videofluoroscopic or fiberoptic endoscopic evaluation is not readily available, such as bedside assessments, community-based dysphagia screening, or home rehabilitation programs. This approach may be particularly useful in settings where VFSS is not readily available or feasible.

### Limitation

This study had several limitations. First, the small sample size limited the generalizability of our findings. Second, the patient population was heterogeneous in terms of underlying medical conditions, which may have influenced the results. Third, although previous studies have suggested that thicker consistencies are associated with greater post-swallow residue, this study evaluated only fluid and yogurt consistencies [24]. Solid foods or materials with varying viscosities were not included in the protocol, which may have limited our ability to assess residue across a broader range of swallowing challenges.

## Conclusions

This pilot study demonstrated that analyzing the HNR measured using an acoustic sensor attached to the vallecular area, either in the pre-swallow state or after YP ingestion, is the most appropriate approach for detecting post-swallow residue. Although a multisite approach was used in this study, the findings suggest that a single vallecular sensor combined with HNR analysis may provide a clinically feasible and simplified method for detecting post-swallow residue. Further largescale studies are warranted to validate these results, refine sensor placement protocols, and explore the integration of this approach into real-time dysphagia assessment systems.
